# Modeling and Studying Acceleration-Induced Effects of Piezoelectric Pressure Sensors Using System Identification Theory

**DOI:** 10.3390/s19051052

**Published:** 2019-03-01

**Authors:** Fujing Xu, Tiehua Ma

**Affiliations:** 1 Department of Automation, Shanxi University, Taiyuan 030013, China; 2 Key Laboratory for Instrumentation Science & Dynamic Measurement, Ministry of Education, North University of China, Taiyuan 030051, China; matiehua@nuc.edu.cn

**Keywords:** pressure sensor, acceleration-induced effects, finite element analysis, system identification

## Abstract

Transient pressure testing is often accompanied by shock acceleration. Aiming at the acceleration-induced effects of pressure sensors, a dynamic compensation method combining empirical mode decomposition (EMD) with system identification theory (SIT) is proposed in this paper. This method is more effective at reducing the error of the acceleration-induced effects without affecting the sensor’s sensitivity and inherent frequency. The principle and theoretical basis of acceleration-induced effects is analyzed, and the static and dynamic acceleration-induced effects on the quartz crystal of a piezoelectric pressure sensor are performed. An acceleration-induced effects dynamic calibration system is built using a Machete hammer, which generates acceleration signals with larger amplitude and narrower pulse width, and an autoregressive exogenous (ARX)mathematical model of acceleration-induced effects is obtained using empirical mode decomposition-system identification theory (EMD-SIT). A digital compensation filter for acceleration-induced effects is designed on the basis of this model. Experimental results explain that the acceleration-induced effects of the pressure sensor were less than 11% after using the digital compensation filter. A series of test data verify the accuracy, reliability, and generality of the model.

## 1. Introduction

In many science and engineering applications, including the measurement of shock waves, compression stress near the projectile base, and pressure of combustion chambers, dynamic transient pressure is tested. Transience is a universality of these pressure signals, which are usually accompanied by huge shocks [1]. The problem is that the effects of the pressure sensor caused by acceleration (acceleration-induced effects) will lead to a large margin of measurement error. Consequently, it is meaningful to explore the acceleration-induced effects of pressure sensors for higher measurement accuracy and precision [2,3].

Piezoelectric pressure sensors are widely used in various pressure tests because of their simple structure, high sensitivity, and high precision. However, the nature structure of piezoelectric sensors makes them sensitive to acceleration, especially to axial acceleration. To measure transient pressure accurately, piezoelectric pressure sensors must compensate for the acceleration-induced effects. Typical methods to eliminate the acceleration-induced effects mainly focus on reducing the mass of the piezoelectric element or improving the mechanical rigidity of the piezoelectric element [4,5]. This inevitably leads to a decrease in the sensitivity of the sensor. For keeping the sensitivity of the sensor constant, an increasing compensation element of the pressure sensor is presented [6]. Nevertheless, this method reduces the inherent frequency of the sensor [7]. The acceleration-induced effect of the pressure sensor was statically calibrated by Zu et al. [8]. However, to eliminate the acceleration-induced error, the acceleration signal must be measured at the meantime, which makes the test system too complicated. Fortunately, this problem can be solved by designing proper digital filters. Hence, a reliable mathematical model might be a big challenge. Shang et al. adopted an empirical mode decomposition (EMD) method to model the acceleration-induced error of piezoelectric sensor [9]. The EMD has many advantages, such as it is intuitive and can be deduced without a preset basis function. In addition, the random noises mixed in the output signal of pressure sensors can be eliminated effectively. Due to a lack of theoretical foundation, the empirical mode decomposition for acceleration-induced effects still has shortcomings in accuracy and reliability.

For these reasons, this paper proposes a new method to compensate the acceleration-induced effects of pressure sensors. The theoretical foundation of acceleration-induced effects is analyzed, and an ANSYS simulation of acceleration-induced effects is performed. Then, an acceleration-induced effects dynamic calibration system is built using a Machete hammer and a series of tests for a piezoelectric pressure sensor are carried out. On the basis of this, a mathematical model is obtained in conjunction with measured data by using empirical mode decomposition (EMD) and system identification theory (SIT). First, the EMD is used to reconstruct the de-noised output of pressure sensors. Second, a suitable mathematical model with optimal parameters is established with SIT. Lastly, a digital compensation filter based on this model is designed to compensate the acceleration-induced effects of pressure sensors. Through the proposed filter, the acceleration-induced effects can be well filtered out. On one hand, this method does not affect the sensor’s sensitivity and natural frequency. On the other hand, EMD-SIT is more effective than the EMD at reducing the error of the acceleration-induced effects.

## 2. Theoretical Model for Acceleration-Induced Effects of Pressure Sensors

**Figure 1:**
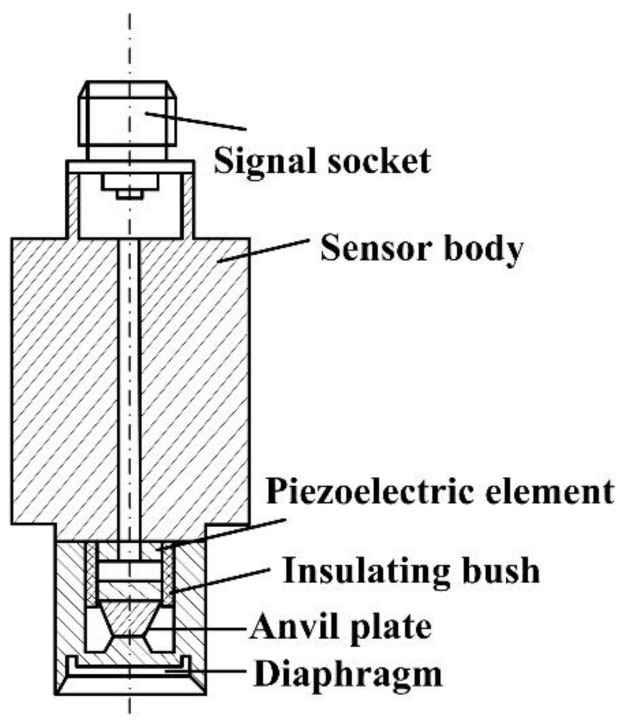
Structure of piezoelectric pressure sensor.

Because of the lack of theoretical basis, the acceleration-induced effects cannot be effectively corrected. Piezoelectric pressure sensors are generally composed of piezoelectric crystal, diaphragm, anvil plate, and other components [10], as shown in Figure 1. The diaphragm delivers pressure and preloads the crystal. When the diaphragm is under pressure, the pressure is passed to the crystal through the anvil plate, which ensures that the pressure on the crystal itself is uniform and prevents local damage from pressure concentrations [11]. 

**Figure 2:**
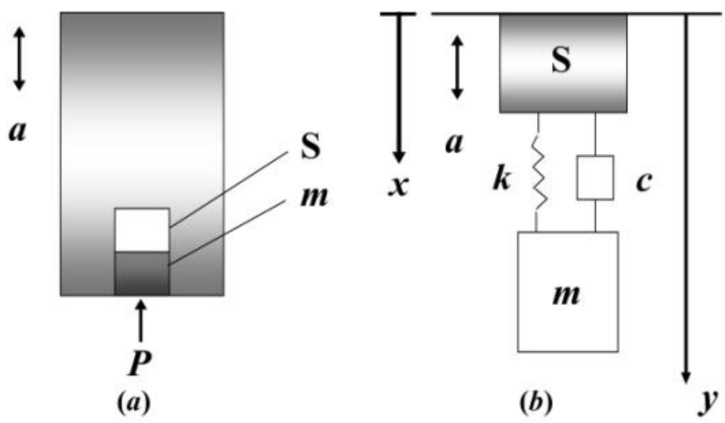
(**a**) Working principle diagram. (**b**) Mechanics principle diagram.

For the purpose of theoretical research, the pressure sensor is equivalent to the model shown in Figure 2a, where *m* is the equivalent mass of the diaphragm, and S is the piezoelectric element.

Figure 2b shows the mechanics principle diagram, where *k* and *c* are the piezoelectric element axial stiffness and damping coefficient, respectively. The *x* and *y* are the geodetic stationary coordinates and internal relative motion coordinates, respectively. From mechanics principles, we obtain the following equation:

(1) $$m\frac{d^{2}y}{dt^{2}}+c\frac{dy}{dt}+ky=-m\frac{d^{2}x}{dt^{2}}$$

where $a(t)=\frac{d^{2}x}{dt^{2}}$ Then, the Laplace transform of Equation (1) is:

(2) $$\left(ms^{2}+cs+k\right)Y=-mA$$

where *Y* is Laplace transform of *y*(*t*), and *A* is Laplace transform of *a*(*t*). From Equation (2), it can be seen that acceleration signals do affect the pressure sensor. We may set $w_{n}=\sqrt{\frac{k}{m}}$ as the undamped natural angular frequency, with $\varepsilon =\frac{c}{2\sqrt{km}}$ as the damping ratio. Therefore, the system transfer function can be written as:

(3) $$H(s)=\frac{-1}{s^{2}+2\varepsilon w_{n}s+w_{n}^{2}}$$

The output charge of the piezoelectric element is proportional to *y*(*t*). Thus, Equation (3) describes the input–output relationship of the pressure sensor and the theoretical model of the acceleration-induced effect of pressure sensor is a second-order linear system. The corresponding amplitude–frequency and phase–frequency characteristics can be described as:

(4) $$A(w)=\frac{\frac{1}{w_{n}^{2}}}{\sqrt{{\left[1-{\left(\frac{w}{w_{n}}\right)}^{2}\right]}^{2}+{\left(2\varepsilon \frac{w}{w_{n}}\right)}^{2}}}$$

(5) $$\phi (w)=\pi -arctg\frac{2\varepsilon \left(\frac{w}{w_{n}}\right)}{1-{\left(\frac{w}{w_{n}}\right)}^{2}}$$

## 3. ANSYS Analysis of Acceleration-Induced Effects of Piezoelectric Element

The sensitive unit of piezoelectric pressure sensors is the piezoelectric element. When the piezoelectric element is subjected to an external pressure and deforms in a given direction, the material surface produces an opposite charge because of polarization. Once the external pressure disappears, the material returns to the uncharged state. If the external pressure changes direction, the polarity of the charge is also changed. This phenomenon is the measurement principle of a piezoelectric pressure sensor [12].

The polarization charge can be calculated as Ref. [13]:

(6) Q=dF

where *d* is the piezoelectric coefficient, and *F* is the pressure. There are many types of piezoelectric materials, such as piezoelectric ceramics, polyvinylidene fluoride (PVDF), quartz crystal, etc. However, quartz crystal has the advantages of stable piezoelectric performance, high mechanical strength, and high insulation impedance, and is commonly used as the sensitive unit of piezoelectric pressure sensor [14]. For quartz crystals, the piezoelectric coefficient matrix can be simplified into a 3 × 6 matrix as follows:

(7) $$d_{ml}=\left[\begin{array}{cccccc} d_{11} & d_{12} & d_{13} & d_{14} & d_{15} & d_{16}\\ d_{21} & d_{22} & d_{23} & d_{24} & d_{25} & d_{26}\\ d_{31} & d_{32} & d_{33} & d_{34} & d_{35} & d_{36} \end{array}\right]$$

The coefficients for quartz crystals in the *x*-direction are shown in Table 1.

**Figure 3:**
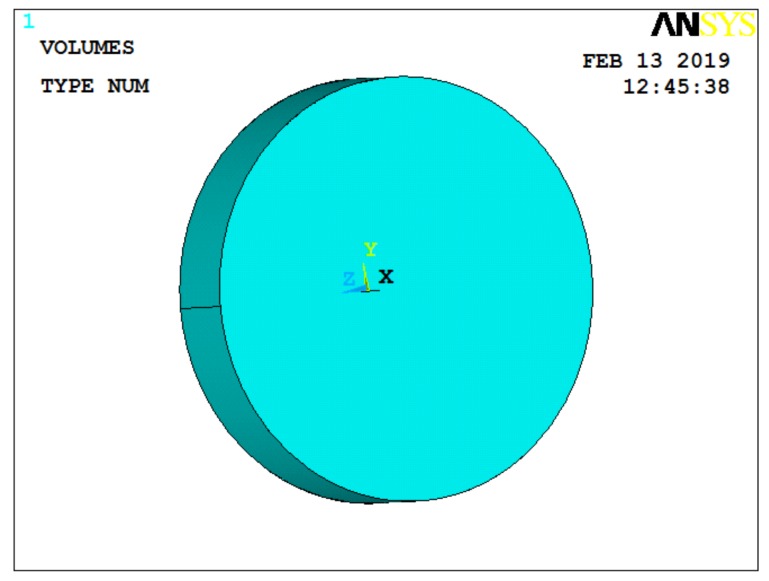
ANSYS model of quartz crystal.

Section 2 indicates the acceleration-induced effect on pressure sensor theoretically. In general, insulation pads and the sensor body of sensors have an insignificant influence on acceleration-induced effects; the diaphragm of sensor is therefore considered when building an ANSYS simulation for the sensor [15,16]. Since the effect of the diaphragm on the quartz crystal can be equivalent to a pressure, we choose only quartz crystal for building the model. The model radius was 9 mm and the thickness was 1 mm, as shown in Figure 3. The finite element type of the model was selected as Solid 5.

**Figure 4:**
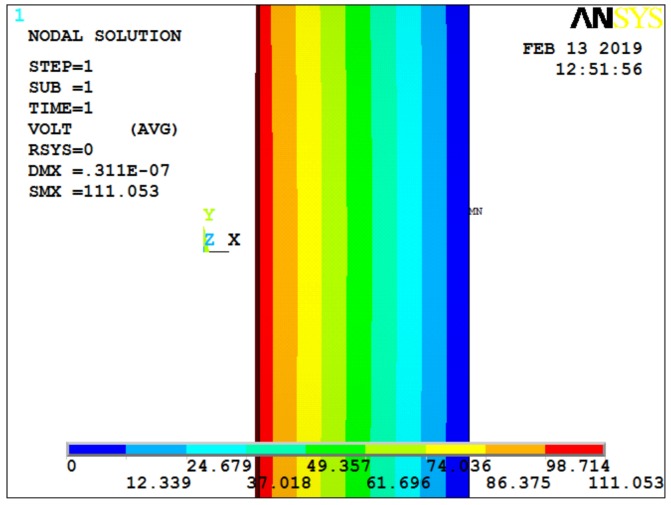
Potential diagram of quartz crystal under 2 MPa of pressure.

**Figure 5:**
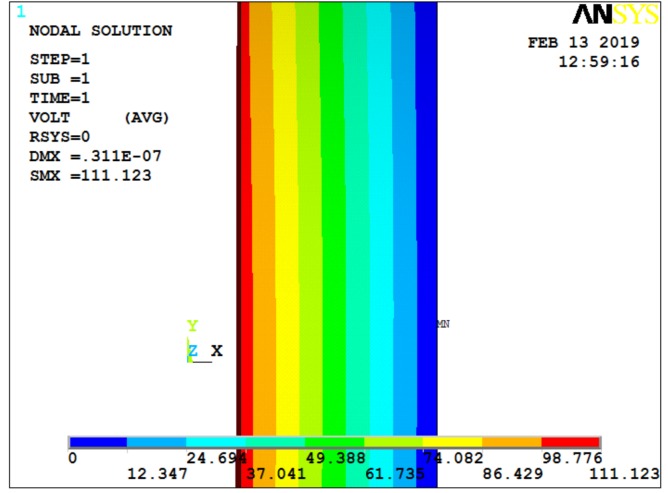
Potential diagram of quartz crystal under 2 MPa of pressure and 1000× *g* static acceleration.

**Figure 6:**
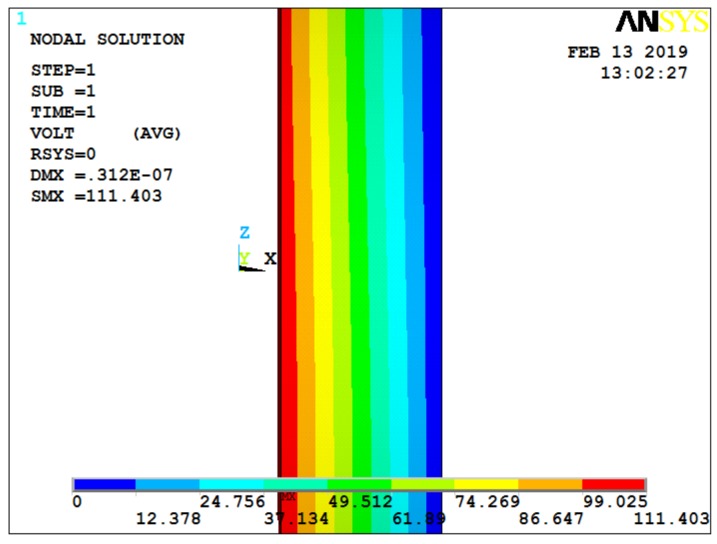
Potential diagram of quartz crystal under 2 MPa of pressure and 5000× *g* static acceleration.

Under this condition, the output result of the quartz crystal is shown in Figure 4, with a 2 MPa pressure exerted on the ANYSYS model. Considering the effect of acceleration on the quartz crystal, Figure 5 and Figure 6 show the cases for additional 1000× and 5000× *g* static acceleration, respectively [17]. Since potential difference is often used to analyze data, the following figures give the ANSYS simulation results of potential.

**Figure 7:**
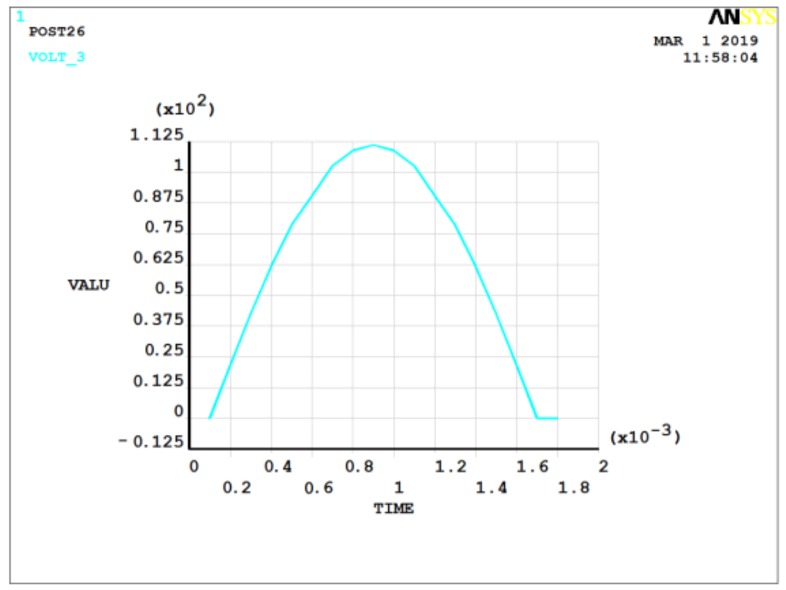
Potential diagram of quartz crystal under 2 MPa of pressure and 1000× *g* dynamic acceleration.

**Figure 8:**
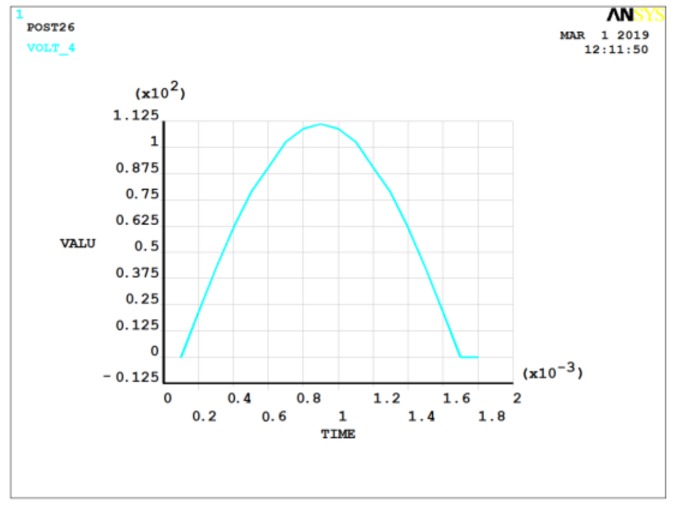
Potential diagram of quartz crystal under 2 MPa of pressure and 5000× *g* dynamic acceleration.

In practical applications, the acceleration signals are mostly dynamic signals with a mathematical form that is usually a half-sine-wave form. Figure 7 and Figure 8 show the case for 2 MPa of pressure applied with 1000× and 5000× *g* dynamic acceleration, respectively. The pulse width of acceleration signals was 1.6 ms.

The ANSYS simulation results show that quartz crystal had an obvious potential difference output under the action of acceleration signals. The relationship between electric potential difference and charge is shown in Equations (8) and (9) [18]:

(8) $$C=\frac{\varepsilon_{0}\cdot \varepsilon_{r}\cdot A}{d}$$

(9) Q=C×U

The output charge was 9.4 × 10^−13^ C under 1000× *g* static acceleration signal action and 4.8 × 10^−12^ C under 5000× *g* static acceleration. In general, the output increased with the acceleration signal increase. For the same acceleration amplitude, the output charge of the dynamic load was generally larger than that of the static load. For example, the output charge was 9.4 × 10^−13^ C under 1000× *g* static acceleration signal action, while that of 1000× *g* dynamic acceleration signal action was 9.6 × 10^−13^ C. Actually, the quartz crystal of piezoelectric pressure sensor was usually thicker than the ANSYS model, which produced a more significant acceleration-induced effect.

## 4. System Modeling and Analysis

### 4.1. Experiments and Results

**Figure 9:**
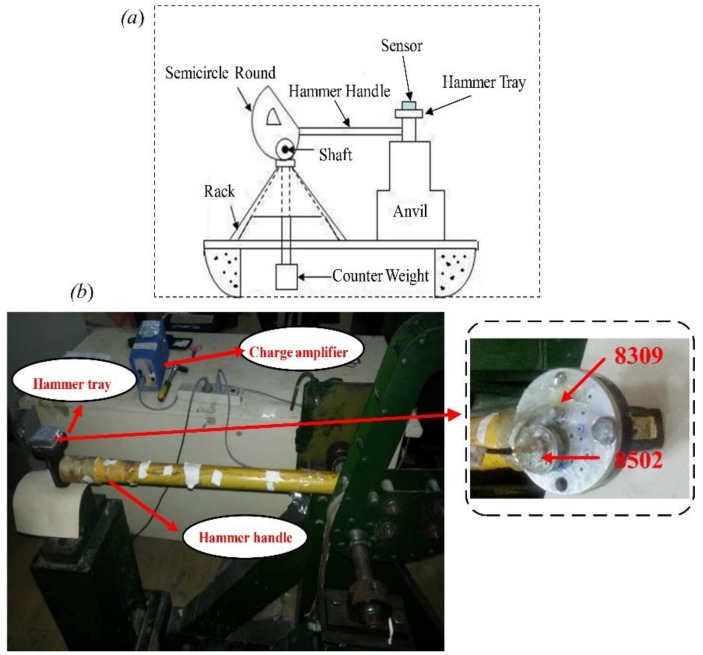
(**a**) Machete hammer principle diagram. (**b**) Test system.

The key of establishing a reliable dynamic mathematical model to describe acceleration- induced effects of pressure sensor is the accurate experimental data. As a common excitation device, a Machete hammer is able to generate a half-sinusoidal acceleration signal with a near millisecond pulse width, which reliably guarantees the transient of the pressure signals [19]. An experimental system for acceleration-induced effects of pressure sensor based on a Machete hammer is designed in this paper. The Machete hammer is composed of hammer, anvil, semicircle round, and counter weight, among others. Figure 9 shows the principle diagram of a Machete hammer. A half-sinusoidal acceleration signal is produced when the hammer hits the anvil. The indicator on the semicircle round shows the magnitude of the acceleration. Apparently, the higher the hammer rises, the larger the acceleration amplitude will be. By adjusting the thickness of the felt between the hammer and the anvil, the pulse width of the acceleration signal can be changed. 

The 8502 piezoelectric pressure sensor, produced by Qi Shi Yuan (Mianyang, China), is widely used in the transient pressure testing. Therefore, it was selected as the test pressure sensor. The 8309 piezoelectric accelerometer (Kistler, Switzerland) which has a sensitivity of 0.04 pC/*g* was selected to measure the additional acceleration. During the test, the 8502 and 8309 sensors were rigidly fixed in the Machete hammer. When the acceleration signal was generated, the output charge of the two sensors were converted into voltage signals by the Kistler charge amplifier 5011, which had a 3 dB bandwidth of 200 KHz, then a high-speed multi-channel oscilloscope whose sampling frequency of data acquisition was 100 MHz, and was adopted to record the pressure and acceleration signal data. The experimental outcomes are detailed in Table 2.

For a constant felt, as the acceleration signal increased, pulse width narrowed, and output charge and pressure amplitude increased. When the acceleration signal was constant, as the thickness of felt increased, pulse width, charge, and pressure amplitude decreased.

In the context of engineering measurements, 1000× *g* is not a large acceleration. However, experimental results indicated that the acceleration-induced effect for the pressure sensor was already 0.1402 MPa, with a corresponding output charge of 14.33 pC, i.e., the acceleration sensitivity of pressure sensor was approximately 0.014 pC/*g*.

**Figure 10:**
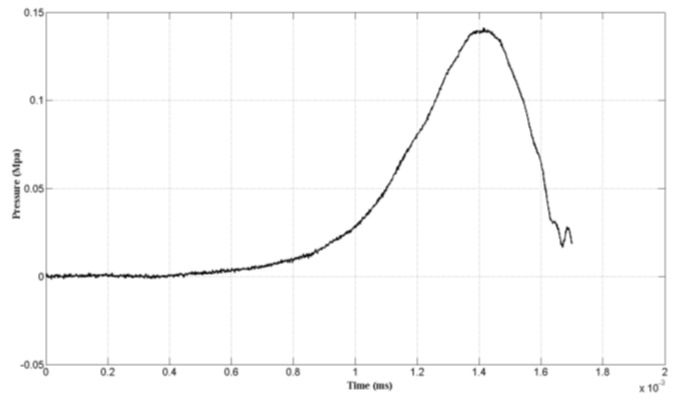
Pressure test data.

A set of Machete hammer test data was selected to analyze. The acceleration acceleration-induced effect of the pressure sensor is shown in Figure 10. The maximum pressure of the signal was 0.1402 Mpa while the pulse width was 1.13 ms.

### 4.2. Modeling and Analysis

With the interference of the various random noise, the output of pressure sensor is usually disordered and unreliable models often occur if directly using the original output for modeling. Therefore, EMD is used to remove the noise from the original signals, which decompose the original output into some specific components with different frequency bands. Considering environmental factors, the relationship between output signal and acceleration is not usually linear. The acceleration-induced effects of a pressure sensor can be modeled using an ARX model [20]:

(10) $$A(d^{-1})y(k)=B(d^{-1})u(k)+\varepsilon (k)$$

or using an autoregressive moving average model (ARMAX) model:

(11) $$A(d^{-1})y(k)=B(d^{-1})u(k)+C(d^{-1})\varepsilon (k)$$

where *u*(*k*) represents the input measured acceleration, *y*(*k*) represents the output pressure, * ε*(*k*) is the random white noise, *n* is the model order, and *d*^−1^ is the backward shift operator, i.e.,

(12) $$A(d^{-1})=1+a_{1}d^{-1}+a_{2}d^{-2}+\ldots +a_{n}d^{-n},\;B(d^{-1})=1+b_{1}d^{-1}+b_{2}d^{-2}+\ldots +b_{n}d^{-n},\;C(d^{-1})=1+c_{1}d^{-1}+c_{2}d^{-2}+\ldots +c_{n}d^{-n}$$

*u*(*k*) and *y*(*k*) are obtained from experimental measured data. We model the acceleration curve using an ARX or ARMAX model. To overcome the shortcomings of the least square method, the generalized least squares method is used to estimate optimal model parameters [21]. The steps to calculate the model parameters are given as follows [22]:

Step 1: Based on the input measured acceleration *u*(*k*) and the output pressure *y*(*k*), the least square estimation forms the initial values, i.e.,

(13) $$a^{\left(1\right)}={\hat{a}}_{LS},\;{\hat{b}}^{\left(1\right)}={\hat{b}}_{LS},\;{\hat{c}}^{\left(1\right)}={\hat{c}}_{LS.}$$

Step 2: In the *l*th iteration, the model parameters are written as:

(14) $${\hat{a}}^{\left(l\right)}={\left[{\hat{a}}_{0}^{\left(l\right)}{\hat{a}}_{1}^{\left(l\right)}\ldots {\hat{a}}_{n}^{\left(l\right)}\right]}^{T},\;{\hat{b}}^{\left(l\right)}={\left[{\hat{b}}_{0}^{\left(l\right)}{\hat{b}}_{1}^{\left(l\right)}\ldots {\hat{b}}_{n}^{\left(l\right)}\right]}^{T},\;{\hat{c}}^{\left(l\right)}={\left[{\hat{c}}_{0}^{\left(l\right)}{\hat{c}}_{1}^{\left(l\right)}\ldots {\hat{c}}_{n}^{\left(l\right)}\right]}^{T}$$

Accordingly, ${\tilde{y}}^{\left(l\right)}(k)$ and ${\tilde{u}}^{\left(l\right)}(k)$ are calculated by:

(15) $$\{\begin{array}{c} {\tilde{y}}^{\left(l\right)}\left(k\right)=y\left(k\right)/{\hat{A}}^{\left(l\right)}\left(d^{-1}\right)\\ {\tilde{u}}^{\left(l\right)}\left(k\right)=u\left(k\right)/{\hat{A}}^{\left(l\right)}\left(d^{-1}\right) \end{array}$$

Subsequently, $\left\{{\tilde{u}}^{\left(l\right)}(k),{\tilde{y}}^{\left(l\right)}(k),k=0,1,\ldots ,N_{0}\right\}$.

Step 3: Update ${\hat{a}}^{\left(l+1\right)}$, ${\hat{b}}^{\left(l+1\right)}$ and ${\hat{c}}^{\left(l+1\right)}$ by performing a least squares method for the following equation.

(16) $$A\left(d^{-1}\right){\tilde{y}}^{\left(l\right)}\left(k\right)=B\left(d^{-1}\right){\tilde{u}}^{\left(l\right)}\left(k\right)+C\left(d^{-1}\right)\varepsilon \left(k\right)$$

**Figure 11:**
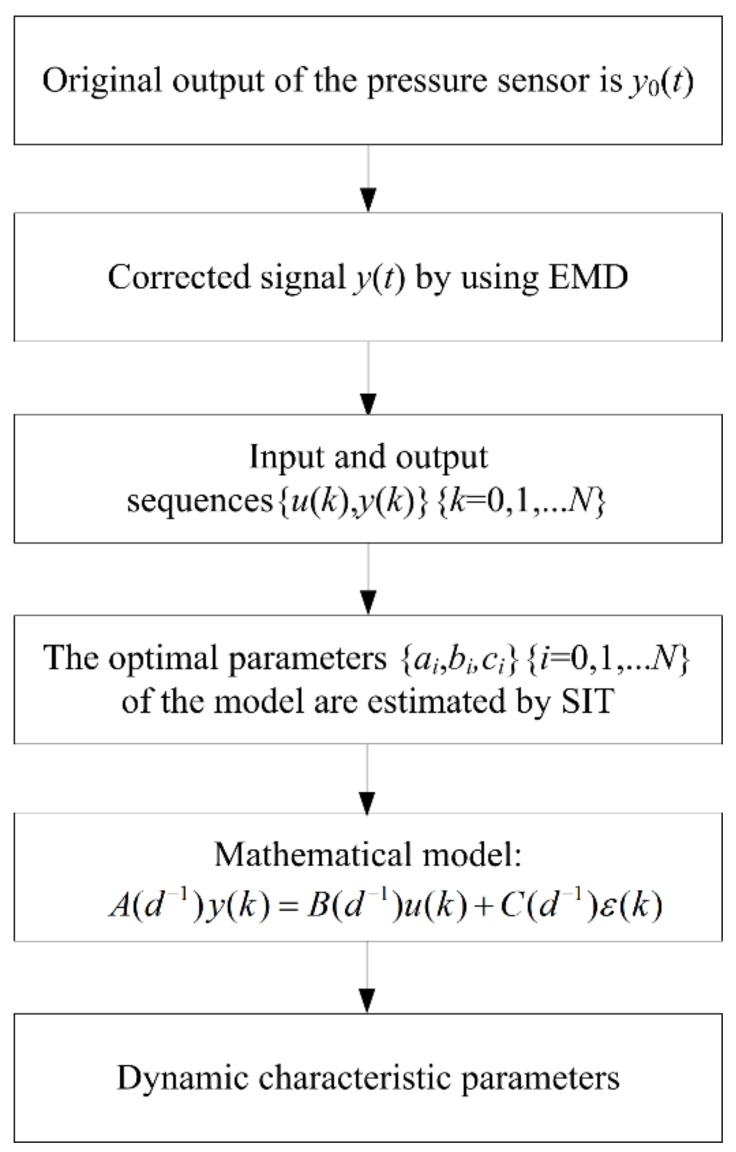
Flowchart of the EMD-SIT.

Step 4: We set *l* = *l* + 1 and repeat Step (2) until the iteration converges or reaches the maximum loops. Figure 11 illustrates the whole flowchart of the EMD-SIT method.

The ARX modeling results are shown in Equations (17) and (18). The system transfer function is shown in Equation (19).

(17) $$A\left(q\right)=1-0.5675\times q^{-1}-0.4075\times q^{-2}$$

(18) $$B\left(q\right)=0.000004037\times q^{-1}$$

(19) $$G(z)=\frac{0.000004037}{z^{2}-0.5675\times z-0.4075}$$

The ARMAX modeling results are shown in Equations (20)–(22). The system transfer function is shown in Equation (23).

(20) $$A\left(q\right)=1-1.889\times q^{-1}+0.8907\times q^{-2}$$

(21) $$B\left(q\right)=0.00000334\times q^{-1}$$

(22) $$C\left(q\right)=1+1.489\times q^{-1}+0.6147\times q^{-2}$$

(23) $$G(z)=\frac{0.000003334}{z^{2}-1.889\times z+0.8907}$$

**Figure 12:**
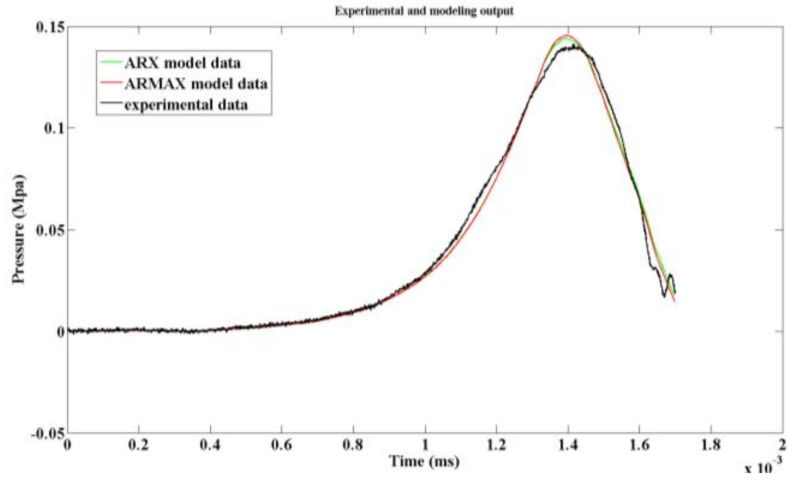
Outputs of the two models.

The modeling results of ARX and ARXMA are both second-order systems. The difference is that the ARMAX model gives a clearer representation of the error *ε*(*k*). Comparatively, the experimental data, the outputs of ARX, and outputs of ARMAX models are shown in Figure 12. 

It can be seen from Figure 12. that the maximum pressure of the ARX model was 0.1448 MPa, while that of the ARMAX model was 0.1439 MPa. The matching rate of ARM model and ARMAX model was 92.72% and 92.57%, respectively, which results in the ARMAX model being slightly more accurate than the ARM model. 

**Figure 13:**
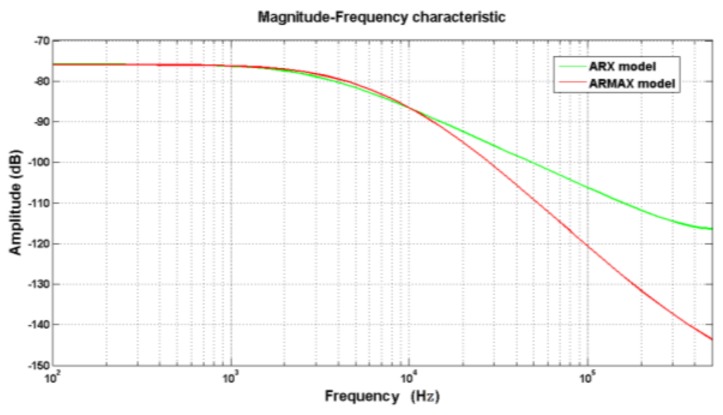
Frequency response of the two models.

**Figure 14:**
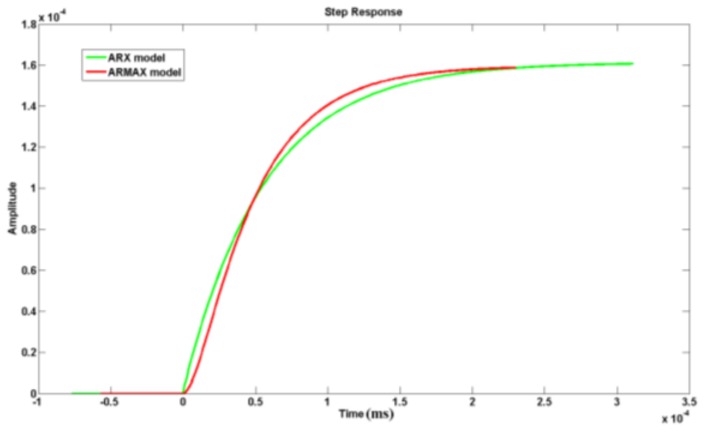
Step response of the two models.

For more characteristics of the models, the frequency response of the two models was calculated as shown in Figure 13. The step response of the two models is shown in Figure 14.

**Figure 15:**
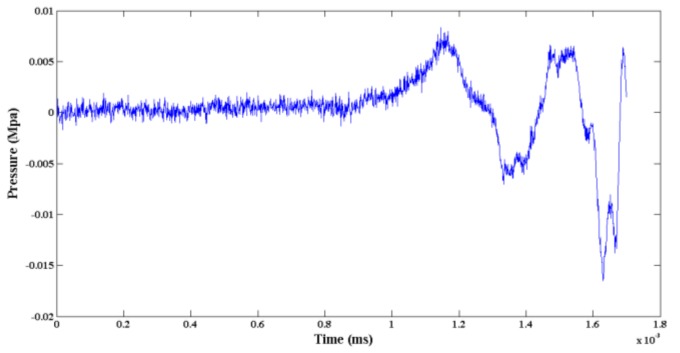
Error curve after compensation.

Comparing the effective bandwidth of the two models, it is clear the ARX had a wider bandwidth. In addition, the step response of ARX model was faster than that of ARMAX model. All these characteristics are crucial for dynamic testing of pressure. In conclusion, to model the acceleration-induced effects of pressure sensors, ARM model was more suitable than the others. Then, a digital compensation filter can be designed according to the ARX model. With the filter in series, the pressure sensor can effectively reduce the acceleration-induced effects. After compensation, the acceleration-induced effects on piezoelectric pressure sensors are shown in Figure 15. 

Figure 15 shows that the pressure maximum of the acceleration-induced effects was reduced to 0.016 MPa, i.e., less than 11%, which illustrates that the characteristics of the acceleration-induced effects could be described perfectly by the ARX model. In order to validate the reliability and universality of the model, acceleration data with different amplitudes were selected for modeling. Excluding certain experimental errors, their mathematical model of acceleration-induced effects was basically consistent. This approach is also applicable to solving the acceleration-induced effects of other pressure sensors. 

## 5. Conclusions

Due to the transient characteristic of transient pressure testing, the acceleration-induced effects of pressure sensors are not negligible. Aiming at offsetting the acceleration-induced effects of pressure sensors, a new dynamic compensation method combining empirical mode decomposition (EMD) with system identification theory (SIT) was proposed. The principle and theoretical basis of acceleration-induced effects was researched, then the static and dynamic acceleration-induced effects on the quartz crystal of a piezoelectric pressure sensor were analyzed by ANSYS. To demonstrate the feasibility of this method, an acceleration-induced effects dynamic calibration system was built using a Machete hammer, which generates acceleration signals with different amplitudes and pulse widths. A series of tests for the 8502 piezoelectric pressure sensor were carried out, and an ARX mathematical model of acceleration-induced effects was obtained using EMD-SIT. A digital compensation filter for acceleration-induced effects was designed on the basis of this model. After compensation, the acceleration-induced effects on pressure sensors were less than 11%. Thus, the correctness, accuracy and reliability of the model were validated. The superiority of our method is that it does not affect the sensor’s sensitivity and inherent frequency. At the same time, EMD-SIT was more effective than the EMD to reduce the error of the acceleration-induced effects. This method is also suitable for many other types of pressure sensors, such as piezoresistive sensors, fiber optic pressure sensors, and so on.

## Figures and Tables

**Table 1:** Coefficient of quartz crystals in *x*-direction.

d_11_ (10^−12^ C/N)	e_11_ (C/m^2^)	g_11_ (m^2^/C)	h_11_ (10^9^ N/C)	s_11_ (10^−12^ m^2^/N)
2.31	0.171	0.0578	4.36	12.77

**Table 2:** Test results of acceleration-induced effects of pressure sensors.

No.	Acceleration (*g*)		Pulse Width (ms)	Output Charge (pC)	Maximum Pressure (MPa)	Mode of Sensor
1	One layer of felt pad	380.9	1.504	6.62	0.07	normal
2		500.6	1.423	8.42	0.089	normal
3		699.3	1.275	10.41	0.11	normal
4		1080	1.044	15.80	0.167	normal
5		1963	0.989	18.28	0.1932	normal
6	Two layers of felt pad	180.4	2.936	3.88	0.041	normal
7		519.7	1.525	8.14	0.086	normal
8		672.5	1.327	10.29	0.1087	normal
9		1006	1.13	14.33	0.1402	normal
10		2185	0.942	18.07	0.191	normal
